# A Critical Review of Effective Child Mass Trauma Interventions: What We Know and Do Not Know from the Evidence

**DOI:** 10.3390/bs11020025

**Published:** 2021-02-11

**Authors:** Betty Pfefferbaum, Pascal Nitiéma, Elana Newman

**Affiliations:** 1 Department of Psychiatry and Behavioral Sciences, College of Medicine, University of Oklahoma Health Sciences Center, P.O. Box 26901, WP3217, Oklahoma City, OK 73126, USA; 2 Department of Management Information Systems, Price College of Business, University of Oklahoma, Norman, OK 73069, USA; pascal.nitiema-1@ou.edu; 3 Dart Center for Journalism and Trauma, Department of Psychology, University of Tulsa, Tulsa, OK 74104, USA; Elana-Newman@utulsa.edu

**Keywords:** anxiety, child, depression, disaster, functional impairment, mass trauma, mental health intervention, political violence, posttraumatic stress, terrorism

## Abstract

Over the last 20 years, numerous interventions have been developed and evaluated for use with children exposed to mass trauma with six publications reporting meta-analyses of randomized controlled trials of child mass trauma interventions using inactive controls to examine intervention effects on posttraumatic stress, depression, anxiety, and functional impairment. The current report reviews the results of these meta-analytic studies to examine the status of the evidence for child mass trauma mental health interventions and to evaluate potential moderators of intervention effect and implications for practice. The meta-analyses reviewed for the current report revealed a small to medium overall effect of interventions on posttraumatic stress, a non-statistically significant to small overall effect on depression, a non-statistically significant overall effect on anxiety, and a small overall effect on functional impairment. The subgroup analyses suggest that interventions should be matched to the populations being served and to the context. Additional research is needed to tailor future interventions to further address outcomes other than posttraumatic stress including depression, anxiety, and functional impairment.

## 1. Introduction

Disasters and political violence, including war, political conflict, and terrorism, have devastating effects on children who are a priority for intervention [1,2]. Children’s mass trauma reactions range from transient emotional distress and behavior changes to enduring psychopathology with as many as 30% experiencing lasting impairment [3]. The most commonly studied disaster outcome is posttraumatic stress disorder (PTSD) and/or PTSD symptoms or reactions [4]. A meta-analysis found that 9.7% of children exposed to non-interpersonal trauma such as accidents and natural disasters developed the condition [5]. Another study found that the risk of lifetime PTSD among adolescents exposed to natural or man-made disasters in the United States was 6.5% [6]. Depression is the second most commonly reported outcome, after posttraumatic stress, with 1.0% to 60% and 1.6% to 33% of children meeting diagnostic criteria for PTSD and depression, respectively [7]. Anxiety, especially anxiety disorders [7], and functional impairment including pervasive and sometimes enduring impairment in emotional, behavioral, cognitive, and social functioning [8] have been less well studied. Factors that influence children’s mass trauma reactions include aspects of the trauma (e.g., type of event, casualty rates, property damage); characteristics of the child (e.g., demographics, pre-existing conditions, prior trauma); exposure of the population, family, and social factors; and contextual factors (e.g., geographic location, available resources) [3]. A meta-analysis of child disaster studies found that posttraumatic stress was more prevalent in girls than boys and that it was associated with disaster-related death rate and with objective and subjective exposure including proximity to the event, personal loss, perceived threat, and distress [4]. 

### Child Mass Trauma Intervention Studies 

Over the last 20 years, numerous interventions have been developed and evaluated for use with children exposed to mass trauma generating systematic qualitative [9,10] and methodological [11,12,13] reviews. More recently, eight publications have reported meta-analyses of randomized controlled trials of child mass trauma interventions including six with studies using only inactive controls [14,15,16,17,18,19] and two with studies using both active and inactive controls [20,21]. These meta-analyses have explored intervention effects on posttraumatic stress [14,17,18,19,20,21], depression [14,15,18], anxiety [15,18], and functional impairment [14,16,18]. Tol and colleagues [19] also examined intervention effects on internalizing symptoms (depression or anxiety). Moderators of intervention effect were explored in some investigations [15,16,17,18,20,21]. The child disaster mental health intervention literature, including meta-analytic studies, is now extensive and warrants a critical review. The purpose of the current report is to review the results of the 12 meta-analyses of randomized controlled trials comparing interventions with inactive controls with the aims of (1) examining the status of the evidence base for intervention and the moderators of intervention effect, (2) considering implications for practice, and (3) identifying issues for future research.

## 2. Materials and Methods

The current review synthesizes the results of 12 meta-analyses of randomized controlled trials using inactive controls to examine posttraumatic stress [14,17,18,19], depression [14,15,18], anxiety [15,18], and functional impairment [14,16,18] outcomes in children receiving interventions for mass trauma. Two publications addressed more than one outcome [14,18]. Morina and colleagues [14] examined posttraumatic stress, depression, and functional impairment, and Purgato and colleagues [18] examined posttraumatic stress, depression, anxiety, and functional impairment [18]. Three of the publications [15,16,17] were authored by the authors of the current review. Because of the high heterogeneity in intervention effects, subgroup analyses were conducted in some of the meta-analytic studies to examine these intervention effects within subgroups that reflect the characteristics of the individual trials. In the current manuscript, the computation of the intervention effect within a subgroup is termed “subgroup analysis”, the trial characteristic (e.g., type of event, population exposure) for which the subgroup analysis was performed is called a “moderator”, and the comparison of intervention effect across subgroups is referred to as “moderator analysis” (e.g., political violence vs. natural disaster, targeted vs. non-targeted sample). Moderator analysis was used to identify factors that explained the variation in results reported by studies with the objective of determining why the effect of the intervention differed across studies. Intervention moderators are reviewed to the extent that they were explored in these meta-analyses [15,16,17,18]. Moderators were categorical variables including event type (e.g., natural vs. manmade), child characteristics (e.g., demographics, exposure), contextual factors (e.g., geographic location, country income level), characteristics of interventions (e.g., individual vs. group), and aspects of intervention delivery (e.g., dose). Effects on outcomes were explored both between (moderator analysis) and within (subgroup analysis) these categories.

## 3. Results

Table 1 presents summary information from the meta-analyses included in this review including the number of trials or studies included in each meta-analysis. 

### 3.1. Differences across Meta-Analyses

**Figure 1:**
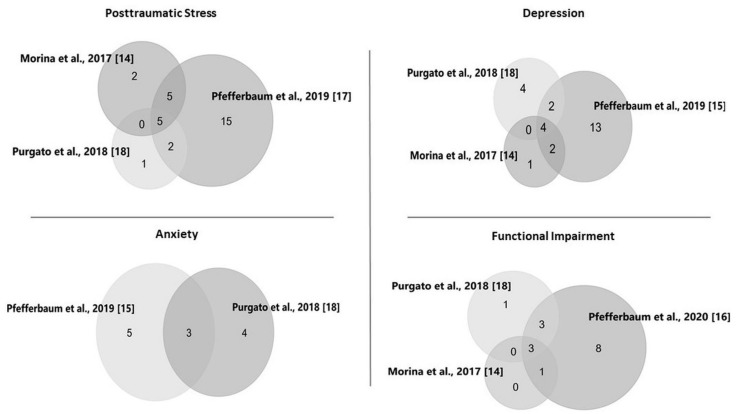
Overlap of trials across the meta-analyses. Note: The numbers in the figures are the numbers of trials that overlap or do not overlap across the meta-analyses. Of the 5 studies included in the meta-analysis of the intervention effect on posttraumatic stress by Tol and colleagues [19], 3 were also included in Morina and colleagues [14], 2 in Purgato and colleagues [18], and 3 in Pfefferbaum and colleagues [17].

The diagram in Figure 1 displays the overlap of trials across meta-analyses by Morina and colleagues [14], Pfefferbaum and colleagues [15,16,17], and Purgato and colleagues [18]. While the general focus was similar across these studies, differences in the goals and the inclusion and exclusion criteria reflect distinct event types, populations, contexts, and intervention characteristics. For example, meta-analyses included trials related to mass violence [14], mass trauma (natural and man-made events) [15,16,17], and traumatic events in humanitarian settings in low- and middle-income countries [18] and in countries where natural or technologic disasters or armed conflict had occurred [19]. Pfefferbaum and colleagues [15,16,17] and Tol and colleagues [19] included trials conducted after natural disasters. Morina and colleagues [14] included only trials conducted in the context of mass violence. Purgato and colleagues [18] included studies of natural disasters in their search, but the only trials selected for their meta-analyses were administered after political violence and war. All of the studies included in the meta-analyses by Morina and colleagues [14], Purgato and colleagues [18], and Tol and colleagues [19] were conducted in low- and middle-income environments, while some of the studies examined by Pfefferbaum and colleagues [15,16,17] were administered in high-income settings. Interventions studied were described as psychological treatment [14], psychological and behavior interventions with no pharmacological component [15,16,17], focused psychosocial interventions characterized as providing emotional and practical support [18], and mental health or psychosocial support practice [19].

### 3.2. Effectiveness of Interventions

As presented in Table 1, the investigation by Morina and colleagues [14], which assessed interventions delivered to young mass violence survivors residing in low- and middle-income countries, found a medium effect for posttraumatic stress in 12 trials, a small effect for depression in 7 trials, and a small effect for functional impairment in 4 trials relative to inactive controls. In meta-analyses of child mass trauma (political violence and natural disasters) intervention studies, Pfefferbaum and colleagues found a medium effect for posttraumatic stress in 27 trials [17]; no statistically significant effect for depression or anxiety in 21 and 8 trials, respectively [15]; and a small effect for functional impairment in 15 trials [16]. Using pooled individual participant data in their investigation, Purgato and colleagues [18] found a small effect for posttraumatic stress in 8 trials; no statistically significant effect for depression or anxiety in 10 and 7 trials, respectively; and a small effect for functional impairment in 7 trials with interventions administered to children in low- and middle-income humanitarian settings. Tol and colleagues [19] found no statistically significant effect for posttraumatic stress in five trials conducted with children living in humanitarian settings (armed conflict and natural and industrial disasters) [19]. See Table 1 for details. 

### 3.3. Follow-Up Findings

Three of the publications reviewed for the current report considered findings at follow-up [14,16,18]. The most extensive analysis, conducted by Purgato and colleagues [18], found that the small beneficial effect on posttraumatic stress assessed within four weeks after administration of the intervention was lower at follow-up six or more weeks after intervention completion and that there was no statistically significant effect on depression and anxiety symptoms after intervention administration or at follow-up. With follow-up assessments ranging from 3 to 12 months, Morina and colleagues [14] found a small effect on functional impairment at both post-intervention and follow-up in their analysis comparing active conditions with waitlist controls. While they did not conduct a formal analysis of follow-up data in their meta-analysis of studies examining functional impairment, Pfefferbaum and colleagues [16] noted that among the included intervention trials that reported follow-up information, the general trend was for a decreased effect over time. 

### 3.4. Moderator and Subgroup Analyses 

Four of the publications reviewed for the current paper reported moderator and subgroup analyses [15,16,17,18] to identify factors that explained the variation in results. Neither Morina and colleagues [14] nor Tol and colleagues [19] reported moderator or subgroup analyses. None of the moderators examined by Pfefferbaum and colleagues for posttraumatic stress [17], depression or anxiety [15], or functional impairment [16] explained the heterogeneity in intervention effect sizes across trials. Purgato and colleagues [18] found significant moderator effects for posttraumatic stress with greater effects in older (15–18 years of age) relative to younger (7–10 years of age and 11–14 years of age) children, in non-displaced relative to displaced children, and in children living in smaller (<6 people) relative to larger (≥6 people) households. Table 2 displays the results of the subgroup analyses with the findings of the moderator analyses in the third column.

These four publications also reported subgroup analyses [15,16,17,18] examining the evidence for intervention effect within subgroups (e.g., event type, sample and/or population characteristics, context, intervention focus, aspects of service delivery). For posttraumatic stress, Purgato and colleagues [18] found evidence of effectiveness for focused psychosocial interventions delivered in low-resource environments across age groups, gender, and geographic region; in children from smaller households (<6 people); and in both displaced and non-displaced children. There was no evidence of effectiveness in children from larger households (≥6 people). Pfefferbaum and colleagues [17] found evidence of effectiveness for posttraumatic stress in targeted (e.g., exposed, at-risk, distressed children) and non-targeted (e.g., universal populations of children regardless of their event exposures, experiences, or reactions) populations exposed to political violence and natural disasters regardless of country income level.

Purgato and colleagues [18] found no evidence of effectiveness with interventions for depression at intervention endpoint across the subgroups they analyzed and evidence of effectiveness for anxiety for children aged 15 to 18 years and for displaced children. Pfefferbaum and colleagues [15] found evidence of improvement in depression with interventions for natural disasters delivered in high-income countries and for interventions that were not trauma-focused and that were delivered in more than eight sessions. For anxiety, Pfefferbaum and colleagues [15] found evidence of effectiveness for anxiety with interventions that were not trauma focused, and Purgato and colleagues [18] found that the intervention effect for anxiety at endpoint was statistically significant for displaced but not for non-displaced children. For functional impairment, Purgato and colleagues [18] found evidence of effectiveness for both boys and girls, for children from small (<6 people) and large (≥6 people) households, for children residing in regions outside Africa but not within Africa, and for displaced and non-displaced children. Pfefferbaum and colleagues [16] found evidence of effectiveness for functional impairment with interventions delivered to targeted populations in low-middle-income countries—but not for interventions delivered to non-targeted samples or for interventions administered in high-income countries—in individual or group applications regardless of the number of sessions (8–12 or >12 sessions). See the first and second columns of Table 2 for details.

## 4. Discussion

The current analysis suggests small [18] or medium [14,17] effects of interventions on posttraumatic stress in children exposed to mass trauma. Tol and colleagues [19] found no effect for posttraumatic stress, but the analysis included only five trials. Morina and colleagues [14] found a small effect for depression while Pfefferbaum and colleagues [15] and Purgato and colleagues [18] found no significant overall effect for depression or anxiety. The effect on functional impairment was small [14,16,18]. Differences in effect sizes across these analyses may reflect differences in the number of included trials and/or in characteristics of the populations, the contexts, the interventions, and/or intervention delivery. The discussion below considers the results of the moderator and subgroup analyses in light of differences across the studies and the implications for practice.

### 4.1. Moderator and Subgroup Findings 

Moderator and subgroup analyses in the meta-analyses reviewed for this report examined characteristics of the event (e.g., type) [15,17] and of the children (e.g., demographics) [18] and populations (e.g., targeted vs. nontargeted) [15,16,17] receiving the intervention; context (e.g., geographic location region of world, income level of country) [15,16,17,18]; and/or aspects of the intervention (e.g., trauma focus vs. no trauma focus, individual vs. group application) [15,16] and/or intervention delivery (e.g., number of sessions) [15,16].

#### 4.1.1. Characteristics of the Event

Consistent with a meta-analysis of posttraumatic stress in children which found comparable effects for natural and man-made disasters [4], event type did not explain the heterogeneity of intervention effects for posttraumatic stress [17], depression [15], or anxiety [15] in the meta-analyses included in this review. Subgroup analyses across the meta-analyses revealed evidence of an intervention effect for posttraumatic stress following either political violence or natural disasters [17] and for depression following natural disasters [15]. Morina and colleagues [14] and Purgato and colleagues [18] included only trials conducted after political violence and war precluding a comparison of the type of event, and while Pfefferbaum and colleagues [16] and Tol and colleagues [19] included trials conducted after natural disasters, neither examined the type of event as a moderator. Other characteristics of the event, such as death toll which appears to influence posttraumatic stress in children [4], may influence intervention effectiveness and warrant further investigation but were not examined in any of the reviewed meta-analyses.

#### 4.1.2. Child Characteristics

Both sociodemographics [18] and population exposure [15,16,17] were examined in the included meta-analyses. 

##### Sociodemographics

Of the meta-analyses that conducted moderator and subgroup analyses, only Purgato and colleagues [18] considered participant sociodemographics (age, gender, and household size), finding a stronger effect on posttraumatic stress for interventions delivered to youth aged 15 to 18 years relative to younger age groups and for children living in households with fewer than six members relative to those living in larger households. None of the sociodemographic moderators explained the observed heterogeneity in intervention effect on depression, anxiety, or functional impairment [18]. Subgroup analyses revealed evidence of intervention effectiveness for posttraumatic stress for both boys and girls in all age groups and for children in households with fewer than six members but not in larger households [18]. Effectiveness for depression was not evident across any demographic variable examined, but there was evidence of intervention effectiveness for anxiety in children 15 to 18 years of age and for functional impairment in both boys and girls in all household sizes [18]. None of the meta-analyses considered the influence of other important child characteristics such as pre-existing conditions and prior or subsequent trauma exposure. Clearly, more work is needed to understand the role of sociodemographic and vulnerability factors in intervention effectiveness across outcomes. 

##### Population Exposure

The type of population exposure (targeted vs. non-targeted) did not explain the heterogeneity among the results reported by the included studies [15,16,17]. There was evidence of effectiveness for posttraumatic stress in both targeted and non-targeted populations [17], no evidence of effectiveness for depression or anxiety overall or in either targeted or non-targeted populations [15], and evidence of effectiveness for functioning in targeted but not non-targeted populations [16]. These discrepancies may reflect the small number of studies in some groups and/or differences in participants’ need for intervention. In general, children in targeted samples were more likely than those in non-targeted samples to have experienced problems warranting intervention and to have high enough symptom levels to reflect improvement post-intervention. Non-targeted populations include children with no or minimal symptoms—some with no need for intervention—which may create a floor effect with relatively low baseline measurement scores and little or no room for improvement. For example, children who display limited transient worry or sadness post-mass trauma should not be expected to demonstrate significant differences pre- and post-intervention.

#### 4.1.3. Contextual Factors

Purgato and colleagues [18] and Pfefferbaum and colleagues [15,16,17] examined contextual factors that may be associated with intervention outcome. The geographic region where the study was conducted as operationalized by Purgato and colleagues [18] did not explain the heterogeneity of intervention effects on posttraumatic stress, depression, anxiety, or functional impairment. The subgroup analyses found evidence of improvement in posttraumatic stress with interventions delivered both within and outside of Africa and of improved functioning in those living outside of Africa but not for those in African regions. They found no evidence of a significant effect on depression or anxiety overall or for either region [18]. While likely used to reflect the cultural, social, and/or economic context, Purgato and colleagues [18] did not explain the rationale for comparing Africa and other regions or discuss their findings on regional differences. Of note, all of the intervention studies included in their meta-analyses were delivered in low-resource environments [18]. 

Of the meta-analyses reviewed for this report, only those by Pfefferbaum and colleagues [15,16,17] included studies conducted in Western and developed countries. The income level of the country where the event occurred or the intervention was delivered represented contextual factors in these meta-analyses [15,16,17], which relied on data from the World Bank to categorize countries as low, middle, or high income based on gross national income per capita. Country income level did not explain the heterogeneity in findings for any of the outcomes examined [15,16,17]. Subgroup analyses revealed improvement in posttraumatic stress at all country income levels [17], improvement in depression in high-income countries [15], and improvement in functioning in low-middle-income countries [16]. 

Exploring another contextual issue, Purgato and colleagues [18] found a statistically significant intervention effect on posttraumatic stress in both displaced and non-displaced children, with the effect being significantly larger in non-displaced children. The intervention effect on anxiety was statistically significant in displaced, but not in non-displaced, children [18]. Displacement effects have important implications for intervention, requiring the examination of numerous related factors. For example, displacement is likely to be more prevalent in those exposed to the harshest of disaster effects, suggesting the potential for severe outcomes. The systematic removal of children out of an affected area may decrease exposure to ongoing danger and hardship and, thus, reduce the severity of their reactions. Natural social support networks may be disrupted for those who are displaced, however, and access to interventions may differ depending on the availability of services post-event.

Though not well investigated, numerous other identifiable social and contextual factors (e.g., population density, cultural and religious practices, preparedness and response infrastructures, social support networks, media consumption) have the potential to influence mass trauma reactions and intervention effectiveness in children. The influence of cultural factors (e.g., disease concepts) also requires further exploration especially given that Western concepts of disorders may not adequately address non-Western psychological and social outcomes and coping [22,23]. The results of future research should guide the development and evaluation of culturally sensitive assessment tools and interventions. 

#### 4.1.4. Intervention Features

The meta-analyses reviewed for this report grouped various intervention types together using global terminology, such as “psychosocial” or “psychological” interventions, to describe the interventions they studied. Specific intervention features such as theoretical orientation, activities, processes, and sequencing were not identified as potential subgroups. While some individual trials included in the meta-analyses described the various components included in their interventions (e.g., psychoeducation, exposure, narrative), for the most part, the extant research has not deconstructed interventions to examine the effectiveness of specific components. In their meta-analyses, Pfefferbaum and colleagues examined two intervention features—trauma focus [15] and individual versus group application [15,16]. Future investigations with greater attention to type of intervention, components, and other features are needed to advance the field. 

##### Trauma Focus 

The medium intervention effect for posttraumatic stress and even smaller effects for other outcomes suggest the importance of considering the appropriate focus of interventions, especially those delivered in low-resource international settings. Posttraumatic stress was the most commonly studied outcome in the meta-analyses included in this review. A review of research on children in the context of complex emergencies noted that while most studies register high rates of PTSD, depression and anxiety may be even more pronounced and may add more to the enduring mental health burden [24]. In addition, ongoing hardships may make depression especially intractable even with intervention [14]. Research conducted in humanitarian settings also recognizes the need to address functioning, skill enhancement, and coping in both the general population and in those with mental health conditions [12]. Although depression was well represented as an outcome across the meta-analyses reviewed for the current report, some of the studies that assessed depression were designed to address posttraumatic stress [14], possibly influencing the meta-analytic outcomes. As evident in Figure 1, considerably fewer studies assessed anxiety, which, along with intervention focus, may account for the non-statistically significant effect for this outcome. Pfefferbaum and colleagues [15] examined the influence of an intervention focus on trauma for depression and anxiety outcomes. 

Because trauma-focused interventions are specifically designed to reduce posttraumatic stress, it is unclear how effective they are in addressing other reactions like depression [14]. This may explain the finding of a small [14] or non-statistically significant overall [15,18] effect on depression and no statistically significant overall effect on anxiety [15,18] in the meta-analyses included in this review. An exemplary study found improvement with a group interpersonal therapy intervention that focused directly on depression symptoms using a culturally sensitive measure of depression [25]. The terminology used to describe the interventions and the components and processes used in the interventions was not consistent across the trials included in the meta-analyses. Thus, the finding by Pfefferbaum and colleagues [15] of non-statistically significant effects for trauma-focused interventions on depression and anxiety should not be considered conclusive. Nonetheless, the findings suggest that trauma-focused interventions may need to be augmented with components specifically directed at depression, anxiety, functioning, and other outcomes, especially important in light of the global health burden associated with depression and anxiety [24]. 

##### Individual versus Group Application 

Both individual and group interventions have been used with children in the context of mass trauma. Subgroup analyses revealed a statistically significant intervention effect for functioning with both individual and group interventions [16], no statistically significant effect for depression with individual or group interventions [15], and no statistically significant effect for anxiety with group interventions [15]. The issue is important for future investigation because group applications, which offer a social component that potentially confers benefit beyond that experienced with individual applications, may be more efficient, cost-effective, and accessible and less stigmatizing than individual applications. 

#### 4.1.5. Aspects of Intervention Delivery 

Like intervention features, specific aspects of intervention delivery may influence outcomes. A qualitative review of youth mass trauma intervention studies described the timing (e.g., disaster phase) and setting (e.g., clinical facilities, schools, community programs) of intervention administration, the training and/or expertise of providers (e.g., mental health professionals, school personnel), and the dose or number of intervention sessions administered [10]. Among the meta-analyses reviewed for the current report, only dose was examined [15,16]. Dose, the amount of the therapeutic agent, can be measured in multiple ways including the quantity or duration of the sessions delivered. Dose is an important issue for consideration especially in resource-poor settings where the number of available providers may be limited [26]. Pfefferbaum and colleagues [15,16] found evidence of effectiveness for depression with interventions delivered in more than eight sessions [15] and on functional impairment regardless of the number of sessions (8–12 sessions or >12 sessions) administered [16]. Unfortunately, measuring dose is complicated because participants may not attend and/or complete the full package of sessions. Because of their implications for service decision making, especially in low-resource environments, intervention dose should be studied further with more precise data, and other aspects of intervention administration (e.g., timing, setting, providers) should be examined.

### 4.2. Implications for Practice

A major issue raised in intervention research is the degree to which the results represent meaningful change with respect to clinical or public health practice. This is especially important for the current analysis because of the small to medium effect sizes in studies that assessed symptom change rather than diagnostic outcomes [14,15,16,17,18,19]. Diagnostic status or symptom severity may reflect a clinically significant outcome in targeted samples of directly exposed and at-risk children. Small levels of symptom reduction may provide great relief to individual children if the baseline level was high and/or the symptoms were particularly problematic. Non-targeted samples typically include asymptomatic or minimally symptomatic children who may not need, or evidence a response to, intervention. In addition, interventions for children in community or distant samples may focus on nonclinical outcomes such as fear, stress, functioning, and wellness as well as posttraumatic stress, depression, and anxiety symptoms [27]. Moreover, to some extent, children’s trauma reactions constitute normal adjustment to extreme events and circumstances rather than manifestations of pathology [28,29]. Thus, studies of children with more severe outcomes may yield larger effect sizes compared to trials that include children with minimal symptom levels at baseline. Additional research is needed to provide a conclusive analysis of symptom reduction and meaningful outcome with regard to children’s symptom levels at baseline and their event exposure. Research is needed as well to identify and/or create tools that establish and ascertain diagnostic and/or meaningful change depending on population characteristics [13]. Some measures of clinical improvement of individuals receiving a psychological intervention have been proposed (e.g., reliable change index [30]) and should be reported routinely in trials that evaluate the effectiveness of interventions addressing disaster-related psychological outcomes.

Another indication of intervention effectiveness is the duration of benefit, which unfortunately has not been well studied. In general, the minimal available data suggest a decrease in effect over time—a finding that should inform decision making regarding whether and when to administer interventions like those reviewed in these studies, especially in low-resource environments. Moreover, the decrease in benefit over time argues for repeated assessment of children especially those with direct exposures and those with clinically significant reactions who may need more intensive treatment and ongoing social support.

### 4.3. Limitations

A number of limitations, in addition to those addressed in the discussion above, warrant attention. Only two teams [15,16,17,18] conducted moderator and subgroup analyses, and, not surprisingly, the two teams explored somewhat different moderators. The moderator and subgroup analyses were limited by information provided in individual studies, which differed considerably in the detail and terminology used to characterize potential moderators. 

Many potentially important moderators were unexplored in the meta-analyses reviewed for the current report, including, for example, those related to the events (e.g., number of casualties, length of conflict), the participants (e.g., pre-existing vulnerabilities), the context (e.g., preparedness and response infrastructures, resources, culture), the interventions (e.g., type, theoretical approach, components, parent involvement, cultural adaptations), intervention administration (e.g., timing, setting, providers), and methodological features (e.g., rating scales, time interval between last session of intervention and post-intervention assessment, analytic strategies). These should be examined in future child disaster mental health intervention studies as should potential interactions among moderators. 

The trials included in the extant evaluation research used group statistical approaches, which may not capture clinical improvement at the individual level [31]. Improved mean scores may conceal harmful effects in some participants [32], especially, perhaps, in non-targeted populations with a range of risks, exposures, and reactions. In addition, the current approach to evidence-based medicine is grounded in randomized controlled trials and meta-analyses that address group outcomes, largely ignoring information about individuals derived from clinical case reports and other approaches to inform good clinical and public health practice [33]. 

Finally, the choice of interventions, populations, and outcomes studied limits the generalizability of the results. For the most part, the included studies did not assess mental health treatment such as psychodynamic psychotherapy and family therapy, and none evaluated psychoactive medications. To the extent that these treatments are used, they should be evaluated in future research. The extant research has largely ignored a determination of the effective components, or active ingredients, of the interventions studied. Much of the focus on children’s mass trauma outcomes, and the focus of many interventions, has been on posttraumatic stress. Greater attention to other outcomes is essential especially in the cross-cultural settings where mass trauma is especially common.

## 5. Conclusions

The current report adds to the literature by reviewing the extant research on child mass trauma intervention effectiveness. With respect to the first aim of the current review to examine the evidence base for intervention, the analysis of the included meta-analyses revealed a small to medium overall effect of interventions on posttraumatic stress, a non-statistically significant to small overall effect on depression, a non-statistically significant overall effect on anxiety, and a small overall effect on functional impairment. The moderator analyses revealed relatively few differences—age, household size, and displacement for posttraumatic stress [18]; geographic region for functional impairment [18]; and intervention focus for anxiety [15]—when contrasting groups on most variables. With respect to the second aim of the current review to consider implications for practice, key findings of the subgroup analyses suggest that decisions about the choice and administration of interventions should match interventions to the populations being served and to the context and should consider the focus of the intervention. With respect to the third aim to identify issues for future research, the current review provides evidence that interventions can be effective for posttraumatic stress outcomes in children exposed to mass trauma. Additional mass trauma interventions for children are needed to address other outcomes including depression, anxiety, and functioning. In addition, further evaluation of the effectiveness of child mass trauma interventions is necessary to determine moderators of intervention effect including characteristics of the event, the children receiving the intervention, the context, the interventions, and intervention administration. The Consolidated Standards of Reporting Trials (CONSORT) [34] has improved reporting qualities on randomized trials and the Preferred Reporting Items for Systematic Reviews and Meta-Analyses (PRISM) [35] has enhanced the ability to report and compare meta-analyses, but standardized terminology and reporting of findings across studies are needed to integrate evidence to guide the development and delivery of child mental health interventions.

## Figures and Tables

**Table 1:** Summary effect sizes of meta-analyses on the effectiveness of interventions on outcomes.

Publication	Number of Trials (k)/Participants (N) ^a^	Summary Effect Size Post-Intervention ^b^	HeterogeneityAmong Studies	Subgroup Analysis	Summary Effect Size at Follow-Up
**Posttraumatic Stress**					
Morina et al. 2017 [14]	k = 12	g = 0.53 (0.25; 0.81)	Not provided	Not conducted	Unclear
Pfefferbaum et al. 2019 [17]	k = 27 N = 4662	g = 0.57 (0.33; 0.81); *p* < 0.0001	Q(26) = 151.21; *p* < 0.0001; I^2^ = 83%	- Traumatic event - Population exposure - Country income	Not conducted
Purgato et al. 2018 [18]	k = 8 N = 2355	SMD = 0.33 (0.14; 0.52); *p* = 0.0006	Q(7) = 35.52; *p* < 0.0001; I^2^ = 80%	- Age - Gender - Household size - Geographic region - Displacement status	6 weeks or later: 0.21 (0.01; 0.42) (k = 6; N = 1808)
Tol et al. 2011 [19]	k = 5 N = 1558	SMD = 0.36 (−0.10; 0.83); *p* = 0.12)	Q(4) = 80.99; *p* < 0.0001; I^2^ = 95%	Not conducted	Not conducted
**Depression**					
Morina et al. 2017 [14]	k = 7	g = 0.25 (0.06; 0.45)	Not provided	Not conducted	Unclear
Pfefferbaum et al. 2019 [15]	k = 21	g = 0.14 (−0.01; 0.28); *p* = 0.0581	Q (20) = 42.63; *p* = 0.0023; I^2^ = 53%; 95% CI = (23%; 71%)	- Traumatic event - Population exposure - Country income - Trauma-focused intervention component - Intervention application (individual vs. group) - Number of sessions	Not conducted
Purgato et al. 2018 [18]	k = 10 N = 2672	SMD = 0.06 (−0.09; 0.21); *p* = 0.44	Q(9) = 32.96; (*p* = 0.0001); I^2^ = 73%	- Age - Gender - Household size - Geographic region - Displacement status	6 weeks or later: 0.09 (0.00; 0.19) (k = 6; N = 1808)
**Anxiety**					
Pfefferbaum et al. 2019 [15]	k = 8	g = 0.39 (−0.07; 0.85); *p* = 0.0855	Q (7) = 27.91; *p* = 0.0002; I^2^ = 75%; 95% CI = (49%; 88%)	- Traumatic event - Population exposure - Country income - Trauma-focused intervention component - Intervention application (individual vs. group) - Number of sessions	Not conducted
Purgato et al. 2018 [18]	k = 7 N = 1969	SMD = 0.03 (−0.13; 0.20); *p* = 0.70	Q(6) = 20.14; *p* = 0.0030; I^2^ = 70%	- Age - Gender - Household size - Geographic region - Displacement status	6 weeks or later: 0.08 (−0.04; 0.19) (k = 4; N = 1264)
**Functional Impairment**					
Morina et al. 2017 [14]	k = 4	g = 0.36 (0.26; 0.49)	Not provided	Not conducted	g = 0.18, 95% CI (0.06; 0.30) (k = 4)
Pfefferbaum et al. 2020 [16]	k = 15 N = 3092	g = 0.33 (0.16; 0.50); *p* = 0.0011	Q(14) = 31.04; *p* = 0.0055; I^2^ = 55% (18%; 75%)	- Traumatic event - Population exposure - Country income - Number of sessions	Not conducted
Purgato et al. 2018 [18]	k = 7 N = 1895	SMD = 0.29 (0.14; 0.43)	I^2^ = 57%	- Age - Gender - Household size - Geographic region - Displacement status	6 weeks or later: 0.09 (−0.05; 0.23) (k = 5; N = 1404)

^a^ The total number of participants is included when reported by the meta-analysis.^b^ The *p*-values for summary effect sizes were not reported by Morina and colleagues [14], g = Hedges’ g; I^2^ = the proportion of variation in effect size estimates that is due to heterogeneity among studies rather than sampling error; k = number of trials; N = total number of participants; Q(df) = the total amount of dispersion among effect size estimates, with df degrees of freedom; SMD = standardized mean difference.

**Table 2:** Results of subgroup analyses conducted across the meta-analyses.

Publication	Subgroups with Statistically Significant Effect Size	Subgroups with Non-Statistically Significant Effect Size	Subgroups with Statistically Significant Difference
**Posttraumatic Stress**			
Morina et al. 2017 [14]	Not conducted	Not conducted	Not conducted
Pfefferbaum et al. 2019 [17]	– Political violence – Natural disaster – Targeted population – Non-targeted population – High-income country – Middle-income country – Low-income country	None	None
Purgato et al. 2018 [18]	– 7–10 years – 11–14 years – 15–18 years – Female – Male – Household size < 6 people – African regions – Regions outside Africa – Displaced (to another village) – Non-displaced (original village)	– Household size ≥ 6 people	– Stronger effect in 15–18 years age group compared to the other age groups – Stronger effect in those from households of <6 people compared to those from households of ≥6 people – Stronger effect in non-displaced subgroup compared to displaced subgroup
Tol et al. 2011 [19]	Not conducted	Not conducted	Not conducted
**Depression**			
Morina et al. 2017 [14]	Not conducted	Not conducted	Not conducted
Pfefferbaum et al. 2019 [15]	– Natural disaster – High-income country – Intervention with no trauma-focused component or process – Intervention with > 8 sessions	– Political violence – Targeted sample – Non-targeted sample – Low-middle-income country – Intervention with trauma-focused component or process – Individual intervention – Group intervention – Intervention with < 8 sessions	None
Purgato et al. 2018 [18]	None	– 7–10 years – 11–14 years – 15–18 years – Female – Male – Household size < 6 people – Household size ≥ 6 people – African regions – Regions outside Africa – Displaced (to another village) – Non-displaced (original village)	None
**Anxiety**			
Pfefferbaum et al. 2019 [15] ^a^	– Intervention with no trauma-focused component or process	– Political violence – Natural disaster – Targeted sample – Non-targeted sample – High-income country – Low-middle-income country – Intervention with trauma-focused component or process – Group intervention – Intervention with < 8 sessions – Intervention with > 8 sessions	– Stronger effect for interventions with trauma-focused component or process compared to interventions with no trauma-focused component or process
Purgato et al. 2018 [18]	– 15–18 years – Displaced (to another village)	– 7–10 years – 11–14 years – Female – Male – Household size <6 people – Household size ≥ 6 people – African regions – Regions outside Africa – Non-displaced (original village)	None
**Functional Impairment**			
Morina et al. 2017 [14]	Not conducted	Not conducted	Not conducted
Pfefferbaum et al. 2020 [16]	– Targeted sample – Low-middle-income country – Individual intervention – Group intervention – Intervention with 8–12 sessions – Intervention with >12 sessions	– Non-targeted sample – High-income country	None
Purgato et al. 2018 [18]	– Female – Male – Household size <6 people – Household size ≥6 people – Regions outside Africa – Displaced (to another village) – Non-displaced (original village)	– 7–10 years – 11–14 years – 15–18 years – African regions	– Stronger effect in regions outside Africa compared to African regions

^a^ Only one trial administered an individual intervention [15]; therefore, the summary effect for individual interventions was not computed by the authors.

## Data Availability

The data described in this manuscript are publicly available and can be found in the five publications selected for this review.
